# Certified

**Published:** 1881-03

**Authors:** 


### CERTIFIED.

At the last annual meeting of the Ohio State Board of Dental Examiners, held in Columbus December 3rd, 1880, the following persons were examined, and, being found qualified, were licensed and so authorized to practice Dentistry in the State of Ohio, according to the provisions of the statute regulating the practice of dentistry, viz.:

Willis D. Lowder, Columbus, O.
A. A. Corbitt, Cincinnati, O.
A. P. Nichols, Chardon, O.
James E. Rose, Salem, O.

